# Dynamic Changes in Membrane Lipid Metabolism and Antioxidant Defense During Soybean (*Glycine max* L. Merr.) Seed Aging

**DOI:** 10.3389/fpls.2022.908949

**Published:** 2022-06-24

**Authors:** Yi-xin Lin, Hai-jin Xu, Guang-kun Yin, Yuan-chang Zhou, Xin-xiong Lu, Xia Xin

**Affiliations:** ^1^National Crop Genebank, Institute of Crop Sciences, Chinese Academy of Agricultural Sciences, Beijing, China; ^2^College of Agriculture, Fujian Agricultural and Forestry University, Fuzhou, China

**Keywords:** artificial aging, glycerolipid, membrane lipid, untargeted lipidomics, antioxidant system

## Abstract

Seed viability depends upon the maintenance of functional lipids; however, how membrane lipid components dynamically change during the seed aging process remains obscure. Seed storage is accompanied by the oxidation of membrane lipids and loss of seed viability. Understanding membrane lipid changes and their effect on the cell membrane during seed aging can contribute to revealing the mechanism of seed longevity. In this study, the potential relationship between oxidative stress and membrane lipid metabolism was evaluated by using a non-targeted lipidomics approach during artificial aging of *Glycine max* L. Merr. Zhongdou No. 27 seeds. We determined changes in reactive oxygen species, malondialdehyde content, and membrane permeability and assessed antioxidant system activity. We found that decreased non-enzymatic antioxidant contents and catalase activity might lead to reactive oxygen species accumulation, resulting in higher electrolyte leakage and lipid peroxidation. The significantly decreased phospholipids and increased glycerolipids and lysophospholipids suggested that hydrolysis of phospholipids to form glycerolipids and lysophospholipids could be the primary pathway of membrane metabolism during seed aging. Moreover, the ratio of phosphatidylcholine to phosphatidylethanolamine, double bond index, and acyl chain length of phospholipids were found to jointly regulate membrane function. In addition, the observed changes in lipid metabolism suggest novel potential hallmarks of soybean seed aging, such as diacylglycerol 36:4; phosphatidylcholine 34:2, 36:2, and 36:4; and phosphatidylethanolamine 34:2. This knowledge can be of great significance for elucidating the molecular mechanism underlying seed aging and germplasm conservation.

## Introduction

Soybean (*Glycine max* L. Merr.) originated in China approximately 5,000 years ago, and currently, it is cultivated worldwide. *Glycine max* L. Merr. is one of the most critical crop legumes, accounting for ∼70% of protein meal consumption and ∼28% of vegetable oil consumption worldwide^1^. However, climate change and population growth require us to breed superiorly performing soybean germplasms to meet the increasing demands for plant proteins, oils, and food. Given the high economic significance and nutritive value of soybean, collecting its germplasm resources and maintaining its genetic diversity have been high priorities for breeders.

Understanding the mechanisms that contribute to seed lifespan and maintaining seed viability during storage are the oldest and most challenging research areas in plant biology. Seed aging eventually leads to the natural and inevitable decline in seed viability, which is affected by genetic variations and environmental influences during seed storage (Ellis et al., 1982; Miura et al., 2002; Walters et al., 2010; Zuo et al., 2018; Colville and Pritchard, 2019; Zhang et al., 2019; Zinsmeister et al., 2020). Prolonged seed storage can trigger a series of biological events, including cellular membrane disruption, nucleic acid modification, DNA and RNA degradation, protein synthesis impairment, and decreased energy metabolism, resulting in loss of seed viability and seed genetic integrity (Kaewnaree et al., 2011; Wang et al., 2015). Therefore, exploring the molecular events associated with seed aging can enhance our understanding of seed longevity during long-term storage.

Reactive oxygen species (ROS) are signaling molecules that play a vital role in plant growth and development, hormone signaling, and response to biotic and abiotic stresses. However, excessive ROS accumulation can cause oxidative stress and induce oxidative damage to nucleic acids, proteins, and polyunsaturated fatty acids (Halliwell, 2006; Bailly et al., 2008; Sharma et al., 2012). Therefore, ROS levels must be strictly balanced between production and scavenging (called the “oxidative window”) to avoid damage to seed viability (Bailly et al., 2008; Rajjou et al., 2012). Enzymatic and non-enzymatic antioxidant systems can effectively scavenge ROS and maintain redox homeostasis (Bailly and Bailly, 2004; Gill and Tuteja, 2010). Antioxidant enzymes involved in antioxidant defense systems include ascorbate peroxidase (APX), catalase (CAT), superoxide dismutase (SOD), monodehydroascorbate reductase (MDHAR), dehydroascorbate reductase (DHAR), and glutathione-reductase (GR) (Rodriguez et al., 2004; Møller et al., 2007). Additionally, ascorbic acid (AsA) and glutathione (GSH) play a crucial part in maintaining cellular redox through non-enzymatic antioxidant homeostasis (You and Chan, 2015). The GSH half-cell reduction potential (*E*_*GSSG/2GSH*_), an assessment index of the cellular redox environment, is significantly negatively correlated with seed viability (Seal et al., 2010; Birtić et al., 2011). Several studies have also found a positive correlation between the reduction in antioxidant system activity and loss of seed viability (Kibinza et al., 2006; Xin et al., 2014; Yin et al., 2014; Xia et al., 2015; Barreto and Garcia, 2017). However, it is unclear how enzymatic and non-enzymatic processes work together to control the redox balance and maintain seed viability in soybean.

Lipids are critical for maintaining life activity as they are implicated in energy metabolism, cell transport, signal transduction, and cytoskeleton composition (Lessire et al., 2009; Okazaki and Saito, 2014). Plants can respond to environmental stress through regulation of the unsaturation and mobilization of phospholipids (Ferreri et al., 2016). According to the structure of the phospholipid classes, phosphatidylcholine (PC), phosphatidylglycerol (PG), and phosphatidylserine (PS) tend to form stable bilayer structures, whereas other phospholipids such as phosphatidylethanolamine (PE) tend to form unstable non-lamellar structures (Cullis et al., 1980; van den Brink-van der Laan et al., 2004). Changes in the carbon chain length of the phospholipid content can also influence membrane structure and function (Bach and Faure, 2010). Several studies have reported that membrane lipid degradation occurs during seed aging (Pukacka and Kuiper, 1988; Ouzouline et al., 2009; Lee et al., 2012; Wang et al., 2012; Oenel et al., 2017). Wang et al. (2012) reported that the degradation of membrane lipids, such as PC and PE, results in phosphatidic acid (PA) formation through phospholipase D (*PLD*) during rice seed aging. Inhibiting *PLDα* activity can help maintain membrane integrity and enhance seed longevity (Devaiah et al., 2007; Lee et al., 2012). Furthermore, downregulation of lipoxygenases, which catalyze the oxidation of polyunsaturated fatty acids, can decrease lipid peroxide content and enhance seed longevity (Bai et al., 2015; Xu et al., 2015; Li et al., 2018). Membrane lipid peroxidation is thus considered a major cause of decreased seed quality and longevity. Nevertheless, how membrane lipid components dynamically change during the seed aging process has been rarely reported to date.

In this study, we explored the effect of antioxidant systems on maintaining the redox balance concomitant with artificial aging treatment in legume soybean seeds and characterized changes in the phospholipid metabolism based on the ultra-performance liquid chromatography (UPLC) quadrupole time-of-flight (QTOF) tandem mass spectrometry MS/MS method. Furthermore, we discussed the relationship between phospholipid change and seed viability and presented novel hallmarks for monitoring seed aging.

## Materials and Methods

### Seed Material and Treatments

Soybean seeds (Zhongdou No. 27) were obtained from the National Crop Genebank of China (Institute of Crop Sciences, Chinese Academy of Agriculture Sciences). The initial seed germination percentage was 99%, and the moisture content was 8.1%. The seeds were maintained for a week at 25°C and 75% relative humidity; consequently, the moisture content reached 12.6% on a fresh weight (FW) basis. The seeds were sealed in an airtight aluminum foil bag, and then they were artificially aged at 40°C for 8 d, 12 d, and 18 d to germination percentages of 85%, 46%, and 20%, respectively. For germination analysis, four replicates of 25 seeds were plated in 9-cm Petri dishes on top of two layers of filter papers that were wetted by 25 mL sterile water and then incubated for 7 d in an artificial climate incubator at 25°C in the dark. Seeds were considered as germinated when the radicle protruded at least 2 mm. Seed imbibition was carried out under the same condition as those for germination. Soybean seed viability was evaluated by the percentage of seed germination after a 7-day germination test according to Xin et al. (2013). The aged seeds were stored at 4°C until further use.

### Measurement of Reactive Oxygen Species, Malondialdehyde, and Electrolyte Leakage

The rate of superoxide radical (O_2_^–^) generation was determined using a nitrous acid assay according to the method of Elstner and Heupel (1976). Embryonic axes (0.1 g FW) were ground in 2 ml of pre-cooled 50 mM sodium phosphate buffer (pH 7.8) in an ice bath. The extracts were centrifuged at 16,000 × *g* for 15 min at 4°C. The supernatant (1 ml) was incubated at 25°C for 30 min with 1 mL of 50 mM sodium phosphate buffer (pH 7.8) containing 2 mM hydroxylamine hydrochloride. Then, the reaction mixture (0.5 mL) was incubated with 0.2 ml of 17 mM sulfanilamide and 0.2 ml of 7 mM 2-naphtylamine at 25°C for 30 min. Then, equal volume of CHCl_3_ was added to the reaction mixture. The supernatant was centrifuged at 3,000 × *g* for 10 min. The absorbance was measured at 530 nm wavelength. A calibration curve was established using sodium nitrite (0–1 μM).

The hydrogen peroxide (H_2_O_2_) content was determined spectrophotometrically as described by Matsubara et al. (1983). The embryonic axes (0.1 g FW) were ground in 1 mL of 5% trichloroacetic acid (TCA) in an ice bath, and the homogenate was centrifuged at 20,000 × *g* for 15 min at 4°C. The supernatant (0.5 mL) was incubated at 25°C for 10 min with 0.05 mL of titanium reagent [20% TiCl_4_ conc. hydrochloric acid (HCl)] and 0.1 mL of 17 M ammonia solution. The precipitate was washed with acetone three times. Finally, the precipitate was dissolved in 2 ml of 1 M H_2_SO_4_, and the absorbance was measured at 410 nm. A calibration curve was generated using H_2_O_2_ (0–1 mM).

The relative electrolyte leakage and malondialdehyde (MDA) levels were used to evaluate the impact of ROS accumulation on membrane integrity in the plant under stress. The concentration of MDA was determined according to the method of Heath and Packer (1968) and Hendry et al. (1992). Embryonic axis samples (0.1 g FW) were homogenized in 2 ml of 10% (w/v) TCA (centrifuged at 12,000 × *g*) for 30 min. The supernatant was collected, and equal volume of 0.67% (w/v) thiobarbituric acid was added. The reaction mixture was boiled for 15 min, instantly cooled in an ice bath, and centrifuged at 10,000 × *g* for 10 min. The absorbance of the supernatant was measured at 450 nm, 532 nm, and 600 nm wavelengths.

Electrolyte leakage was determined according to the method described by Xin et al. (2014). Ten whole dry seeds were soaked in 25 mL of Milli-Q water (Millipore, Milford, MA, United States) at 25°C for 24 h. Conductivity (μS cm^–1^) was determined using a Delta 326 electrical conductivity meter (Mettler Toledo, Columbus, OH, United States). The absolute conductivity was measured after treating the seeds with boiling water for 30 min. The results were presented as relative electrolyte leakage.

### Enzymatic and Non-enzymatic Antioxidant Determination

The antioxidant system of the seed embryonal axis was analyzed using a spectrophotometer. The levels of total ascorbic acid (AsA+DHA) and AsA content in the seed embryonic axes were determined by monitoring the increase in pink complexes from the reaction of Fe^2+^ and bipyridyl at 525 nm (Law et al., 1983). Embryonic axes (0.1 g FW) were homogenized in 3% (w/v) metaphosphoric acid in an ice bath and centrifuged at 12,000 × *g* for 20 min at 4°C. The AsA content was determined at 525 nm using 1 mL reaction mixture containing 5% supernatant (v/v), 2% trichloroacetic acid (w/v), 10% phosphoric acid, 0.8% bipyridyl, and 0.3% FeCl_3_. Total ascorbic acid was determined using the same method at 25°C for 20 min with dithiothreitol to reduce all DHA to AsA before the development of color. A standard curve of AsA was used for quantification (0–1 mM).

Reduced GSH and oxidized GSH (GSSG) contents were determined using the 5,5′-dithiobis-(2-nitrobenzoic acid)-GR recycling procedure according to the method of Griffith (1980). Embryonic axes (0.1 g FW) were homogenized in 5% sulfosalicylic acid in an ice bath and centrifuged at 12,000 × *g* for 20 min at 4°C and the supernatant was collected for analysis of GSH. Total GSH was determined at 412 nm using 1 mL reaction mixture containing 2% supernatant (v/v), 50 mM phosphoric acid buffer (pH 7.5), 1 mM DTNB, 0.2 mM NADPH and 20 μL GR (50 U/mL). GSSG was determined using the same method by using 2-vinylpyridine to derivatize GSH before adding GR. A standard curve of GSH was used for quantification (0–0.1 mM).

The embryonic axes (0.1 g FW) were ground in 1.8 mL of 50 mM pre-cooled Tris–HCl buffer (pH 7.0, containing 5 mM MgCl_2_) in an ice bath. The mixtures were centrifuged at 16,000 × *g* for 20 min at 4°C. The protein content of the supernatant was determined according to the method of Bradford (1976), and then the extracts were used to assay antioxidant enzyme activity. SOD (EC 1.15.1.1) activity was assayed by monitoring the photochemical reduction inhibition of nitroblue tetrazolium (NBT) at 560 nm and 25°C (Beyer and Fridovich, 1987). The reaction mixture contained 50 mM potassium phosphate (pH 7.8), 0.1 mM EDTA, 75 mM NBT, 13 mM methionine, 16.7 mM riboflavin, and 20 μg enzyme protein in a final volume of 1 mL. The mixtures reacted under 2,200 lux fluorescent lamp for 15 min. One unit of SOD was defined as the enzyme activity which inhibited the photo-reduction of NBT to blue formazan by 50%. CAT (EC 1.11.1.6) activity was determined by measuring the decomposition of H_2_O_2_ at 240 nm and 25°C (0.04 mM^–1^ cm^–1^) (Tanida, 1996). The reaction mixture contained 10 mM H_2_O_2_ in 50 mM phosphate buffer (pH 7.0) and 20 μg enzyme protein in a total volume of 1 mL. APX (EC 1.11.1.7) activity was measured as the decrease in the oxidized ascorbate absorbance at 290 nm and 25°C (2.8 mM^–1^ cm^–1^)due to oxidation by H_2_O_2_ (Nakano and Asada, 1981). The reaction mixture contained 50 mM potassium phosphate (pH 7.0), 1 mM ascorbic acid (AsA), 0.25 mM H_2_O_2,_ and 20 μg enzyme protein in a total volume of 1 mL. GR (EC 1.6.4.2) activity, as described by Madamanchi and Alscher (1991), was determined by the decrease in absorbance at 340 nm and 25°C (6.2 mM^–1^ cm^–1^) due to nicotinamide adenine dinucleotide phosphate (NADPH) oxidation. The reaction mixture contained 50 mM potassium phosphate (pH 7.8), 0.5 mM GSSG, 5 mM MgCl_2_, 0.15 mM NADPH, and 20 μg enzyme protein in a total volume of 1 mL. DHAR (EC 1.8.5.1) activity was measured by monitoring the increase in absorbance at 265 nm and 25°C (14 mM^–1^ cm^–1^) caused by DHA formation, as described by Dalton et al. (1993). The reaction mixture contained 50 mM potassium phosphate (pH 6.3), 1 mM oxidized ascorbate (DHA), 2 mM GSH, and 20 μg enzyme protein in a total volume of 1 mL. MDHAR (EC 1.6.5.4) activity was assayed according to Arrigoni et al. (1992) by monitoring the decrease in absorbance at 340 nm and 25°C (6.2 mM^–1^ cm^–1^) due to nicotinamide adenine dinucleotide (NADH) oxidation. The reaction mixture contained 50 mM potassium phosphate (pH 7.0), 0.2 mM NADH, 2.5 mM AsA, 0.2 U ascorbate oxidase, and 20 μg enzyme protein in a total volume of 1 mL.

### Lipid Extraction and UPLC-QTOF-MS/MS Analysis

Lipid extraction and data analysis were performed as described by Sarafian et al. (2014) and Lin et al. (2019). Samples of the embryonal axis, collected at four aging time-points, were grounded into powder in liquid nitrogen. For lipids extraction, 50 mg of embryonal axis powder was mixed with 900 μL of extraction buffer (MS grade chloroform:methanol = 2:1, v/v). After incubation at −20°C for 2 h, the samples were centrifuged for 10 min at 14,000 rpm and 4°C. Thereafter, the supernatant was transferred to a fresh glass vial for lipidomics analysis.

Quality control (QC) samples were prepared by pooling aliquots of all samples that were representative of the samples. Blank samples (extraction buffer) and QC samples were injected at the beginning and after every six samples during acquisition. The extracted samples were re-randomized for LC-MS analysis such that the injection order was independent from the order of sample preparation to minimize systematic bias. The UPLC-QTOF/MS analyses were performed using a UPLC system (ACQUITY UPLC I-Class, Waters, Milford, MA, United States) coupled to an electrospray ionization quadruple time-of-flight mass spectrometer (Xevo G2-S Q-TOF, Waters, Milford, MA, United States). Waters ACQUITY UPLC CSH C18 column (1.7 μm; 100 mm × 2.1 mm) was used for the LC separation, and the column was maintained at 45°C. The flow rate was 0.4 mL/min, and the sample injection volume was 2 μL. The mobile phase A was 0.1% formic acid/10 mM ammonium formate in acetonitrile/water 6:4 v/v and B was 0.1% formic acid/10 mM ammonium formate in IPA/acetonitrile (9:1 v/v). The initial linear gradient was as follows: 40% B, 0–2 min; 40% B to 43% B, 2–2.1 min; 43% B to 50% B, 2.1–12 min; 50% B to 54% B, 12–12.1 min; 54% B to 70% B, 12.1–18 min; 70% B to 99% B, 18–18.1 min; 99% B to 40% B, 18.1–20 min; 40% B. High-accuracy MS data were recorded using MassLynx 4.1 software (Waters, Milford, MA, United States). Capillary voltage was 3 kV for both positive and negative mode, whereas cone voltage was 25 V for both modes. Source temperature was set at 120°C with a cone gas flow of 50 L/h, and desolvation temperature was set at 400°C with desolvation gas flow of 800 L /h. Leucine-enkephalin (Waters, Milford, MA, United States) was used as the lock mass generating a reference ion at m/z 556.2771 in the positive mode and m/z 554.2615 in the negative mode, which was introduced by a locking spray at 5 μL/min for data calibration. The MS^E^ data were acquired in continuum mode using ramp collision energy in two scan functions. For low energy mode, the scan range was 100–1,500 Da, scan time was 0.2 s, and collision energy was 6 V. While for high energy mode, the scan range was 100–1,500 Da, scan time was 0.2 s, and collision energy ramp was 15–60 V.

The screened data were taken into account after correcting for individual bias using QC and blank data. The lipids were annotated using LIPID MAPS^2^ database combined Progenesis QI (version 2.4, Non-linear dynamics, Waters, Milford, MA, United States).

The phospholipid double bond index (DBI) and acyl chain length (ACL) of phospholipids are critical indicators of membrane lipid fluidity.

The DBI was calculated as follows:


DBI=[Σ(N×%phospholipid)]/100,

where N is the number of double bonds. The ACL was calculated using the following formula:


ACL=[Σ(NC×%phospholipid)]/100,

where NC is the number of acyl carbon atoms in each molecular species.

### Statistical Analysis

All statistical analyses were performed using SPSS version 22 (IBM, Armonk, NY, United States). Three replicates were analyzed for determining ROS, MDA, relative electrolyte leakage, and enzymatic and non-enzymatic antioxidants. Six replicates were analyzed for UPLC-QTOF-MS/MS analysis. Pearson correlation analysis was performed based on the two-tailed test. Mean separations were analyzed using one-way analysis of variance by calculating the least significant difference. Differences were considered significant at *p* < 0.05. Principal component analysis (PCA) and partial least-squares discrimination analysis (PLS-DA) were applied to the data using MetaboAnalyst software^3^ to determine the lipid differences. The screening criteria of lipids with significant differences between the embryonic axes of adjacent time points (such as 0 d vs. 8 d, 8 d vs. 12 d, and 12 d vs. 18 d) were set as variable importance in projection (VIP) > 1 and *p*-value < 0.05.

## Results

### Antioxidant System Analysis in Embryonic Axis During Seed Aging

**FIGURE 1:**
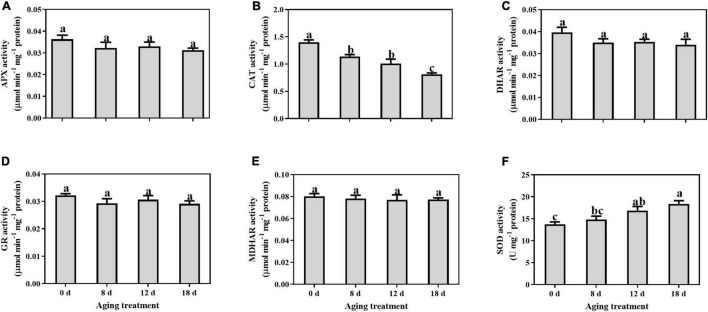
Changes in the activities of **(A)** APX, **(B)** CAT, **(C)** DHAR, **(D)** GR, **(E)** MDHAR, and **(F)** SOD in the embryonic axis during seed aging. Data shows the mean ± SD of three biologically independent experiments. Columns marked with different letters (a-c) are statistically significantly different (*p* < 0.05).

No significant changes were observed in the activity levels of APX, DHAR, GR, and MDHAR, during seed aging (Figures 1A,C–E). The CAT activity level decreased significantly by 16.56%, 28.04%, and 44.50% in the seed embryonic axes aged for 8 d, 12 d, and 18 d, respectively (Figure 1B). In contrast, SOD activity increased gradually but significance was observed in the seed embryonic axes aged 12 d and 18 d (by 22.84% and 33.87%, respectively) (Figure 1F).

**FIGURE 2:**
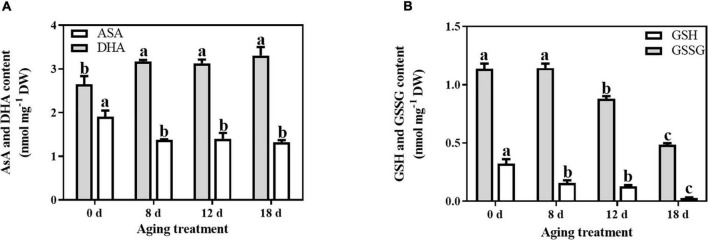
Effects of artificial aging treatment on the **(A)** AsA and DHA contents, and **(B)** GSH and GSSG contents. Data shows the mean ± SD of three biologically independent experiments. Columns marked with different letters (a-c) are statistically significantly different (*p* < 0.05).

In comparison with the changes in antioxidant enzymes, the non-enzymatic antioxidants were significantly affected by the artificial aging treatments. The AsA content decreased sharply by 27.89% after 8 d aging treatment and then remained unchanged (Figure 2A). In contrast, the change in DHA content exhibited the opposite pattern. After 8 d of aging, the DHA content from the embryonic axes of seeds increased significantly by 19.94%. The level of total AsA remained unchanged throughout the whole aging treatment. In contrast, the total GSH content decreased with prolonged aging treatment (Figure 2B). The GSH content was significantly reduced with artificial aging treatment (by 51.47%, 60.86%, and 90.67% in the seed embryonic axes aged 8 d, 12 d, and 18 d, respectively). Additionally, the GSSG content remained unchanged at first and then decreased rapidly by 22.85% and 57.34% at 12 d and 18 d of aging treatment, respectively.

### Reactive Oxygen Species Accumulation and Membrane Lipid Damage During Seed Aging

**FIGURE 3:**
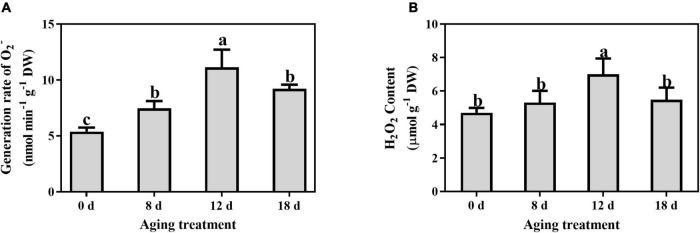
Effects of artificial aging treatment on **(A)** generation rate of O_2_^–^ and **(B)** content of H_2_O_2_. Data shows the mean ± SD of three biologically independent experiments. Columns marked with different letters (a-c) are statistically significantly different (*p* < 0.05).

The O_2_^–^ generation rate and H_2_O_2_ content were determined to investigate the change in ROS concentrations in artificially aged seeds. The generation rate of superoxide anion increased rapidly at 8 d, and a peak was detected after artificial aging for 12 d, followed by a decrease in the seeds aged 18 d (Figure 3A). A similar pattern was observed for the change in H_2_O_2_ content (Figure 3B), which peaked in the seeds aged 12 d.

**FIGURE 4:**
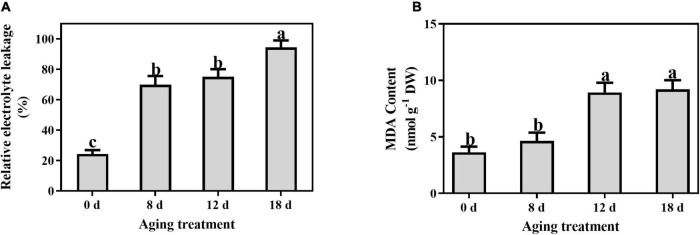
Effects of artificial aging treatment on **(A)** relative electrolyte leakage and **(B)** MDA content. Data shows the mean ± SD of three biologically independent experiments. Columns marked with different letters (a-c) are statistically significantly different (*p* < 0.05).

Compared to the control, the relative electrolyte leakage increased markedly from 21.55% to 69.92% after artificial aging for 8 d and continued to increase with a longer aging duration (Figure 4A). A significant increase in the MDA content was observed in the seeds aged 12 d (from 3.65 ± 0.50 nmol g^–1^ to 8.93 ± 0.86 nmol g^–1^) and then remained unchanged (Figure 4B).

### Modification of Membrane Lipid Composition During Seed Aging

**FIGURE 5:**
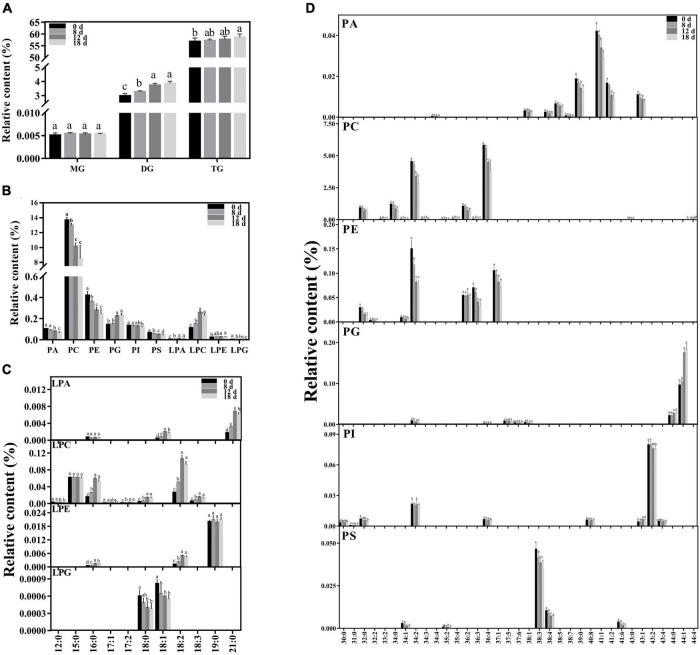
Changes in lipid composition during seed aging. **(A)** Glycerolipid composition changes during seed aging. **(B)** Phospholipid composition changes during seed aging. **(C)** Lysophospholipid composition changes during seed aging. **(D)** Phospholipid molecular species composition changes during seed aging. Data shows the mean ± SD of six biologically independent experiments. Columns marked with different letters (a-d) are statistically significantly different (*p* < 0.05). Lipid molecular species are identified as total acyl carbons: total double bonds.

Approximately 223 lipid molecular species were detected (Figure 5 and Supplementary Table 1), including six categories of phospholipids (PA, PC, PE, PG, phosphatidylinositol (PI), and PS), four categories of lysophospholipids [lysophosphatidic acid (LPA), lysophosphatidylcholine (LPC), lysophosphatidylethanolamine (LPE), and lysophosphatidylglycerol (LPG)], and three categories of glycerolipids [monoacylglycerol (MG), diacylglycerol (DG), and triacylglycerol (TG)].

The relative contents of the glycerolipids exhibited increasing patterns with a rise in the seed aging duration (Figure 5A). The level of MG did not change during this process. However, the relative content of DG increased significantly from 2.98% to 3.85%. Additionally, a higher TG content was only observed in the seeds aged 18 d.

Phospholipids are crucial components of the membrane skeleton (Figure 5B). As the treatment time increased, the level of total phospholipids significantly decreased. Significant decreases in the PC, PE, and PS contents were observed in the seeds aged 8 d, and they were further reduced by 37.56%, 42.08%, and 31.6% after 18 d of aging treatment, respectively. The PA content rapidly declined in the seeds aged 12 d, ultimately decreasing by 35%. The content of PI remained nearly stable in the early stages and decreased by 8.81% in the seeds aged 18 d. In contrast, a significant increase in PG was observed in the seeds aged 12 d. To further explore the changing pattern of the membrane lipid species, we compared the changes in the lysophospholipid and phospholipid species contents during seed aging (Figure 5D). All PA species contents were significantly reduced after 18 d of aging treatment (by 29.05–54.32%), except for PA 34:4. The major contributors to PC degradation were PC 32:0, 34:0, 34:2, 36:2, and 36:4 molecular species, and a rapid decrease was observed in seeds aged 12 d (by 28.81%, 31.1%, 25.65%, 32.13%, and 23.21%, respectively). In addition, the PC 44:4 content increased by 27.56% after 18 days of treatment. PE 34:2, 36:2, 36:3, and 37:1 were the primary PE molecular species, accounting for over 90% of the total PE content. PE 36:3 remained stable during the aging treatment. The PE 34:2, 36:2, and 37:1 content decreased rapidly after 8 d; they were reduced by 50.85%, 53.19%, and 33.04%, respectively, after 18 d of aging treatment. PG 44:0 and 44:1, the major PG species, remained stable during the early aging stage and then increased sharply after 12 d of aging. PI 34:2 and 43:2 were the primary PI molecular species, accounting for over 76% of the total PI content. PI 43:2 exhibited a decreasing trend with aging time, while PI 34:2 demonstrated no apparent change. PS 38:3 and 38:4 accounted for 89% of the total PS content. Both PS 38:3 and 38:4 exhibited a decreasing trend under seed aging and were reduced by 24.07% and 43.29%, respectively, after 18 d of treatment.

Compared with the change in phospholipids, the corresponding lysophospholipids exhibited the opposite change pattern. The contents of LPA, LPC, and LPE increased by 192.76%, 107.9%, and 23.72% after 18 d of treatment, respectively. In contrast, the LPG was reduced by 62.17%. Artificial aging treatment significantly decreased the contents of most lysophospholipid species, including LPA 18:1 and 21:0, LPC 16:0, 17:2, 18:0, 18:2, and 18:3, and LPE 16:0 and 18:2, and the peaks of these lysophospholipid species were found in the seeds aged 12 d. The contents of LPC 12:0 and 17:1 and LPG 18:0 and 18:1 significantly decreased after the aging treatment. The other lysophospholipid species remained stable.

### Changes in Phospholipid Structures Under Seed Aging

**FIGURE 6:**
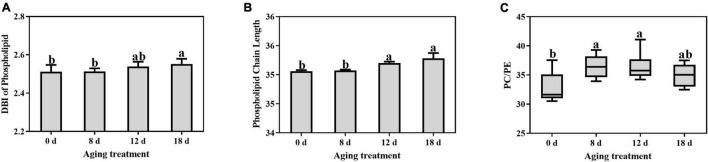
Effects of artificial aging treatment on the phospholipid **(A)** DBI, **(B)** ACL, and **(C)** PC/PE ratio. Data shows the mean ± SD of six biologically independent experiments. Columns marked with different letters (a-b) are statistically significantly different (*p* < 0.05).

The DBI of phospholipids remained unchanged and then increased significantly in the seeds aged 18 d (Figure 6A). The DBI of PC demonstrated the same change pattern (Supplementary Table 2). In contrast, the DBI of PE, PI, PG, and PS significantly decreased within 12 d to 18 d of aging treatment. The ACL of the phospholipids increased rapidly after 12 d of treatment and then continued to increase (Figure 6B). In contrast, there was no change in the PA ACL. Additionally, PC, PE, PI, PG, and PS exhibited significant increases in ACL within 8 d to 18 d of aging treatment (Supplementary Table 3). A significant increase in the PC to PE ratio was observed in seeds aged 8 d (Figure 6C), which indicated that the degradation of the unstable structures of phospholipid species, such as PE, was much earlier than that of stable phospholipids.

### Identification of Critical Lipids Between Adjacent Aging Time Points Using Principal Component Analysis and PLS-DA

**FIGURE 7:**
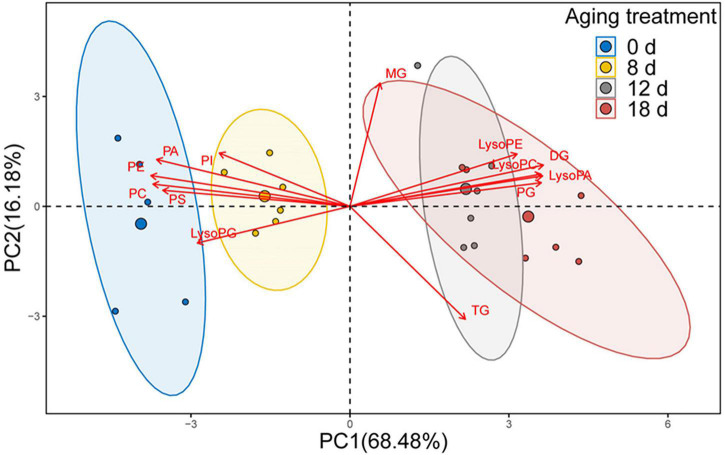
PCA loading biplot illustrating the effect of 13 lipid molecules (PA, PC, PE, PG, PI, PS, LPA, LPC, LPE, LPG, MG, DG, and TG) on seed viability.

We used PCA to analyze the changes in phospholipids, lysophospholipids, and glycerolipids to explore the differences between samples and determine which variables are predominately responsible for the observed differences (Figure 7). The first two principal components accounted for 84.66% of the variance (PC1, 68.48%; PC2, 16.18%). The distribution of the samples within the biplot revealed that each aging timepoint was distinct, except for the samples from the seeds aged 12 d and 18 d. Moreover, the 0-d and 8-d samples were near to each other. We observed that the primary variables were represented by phospholipids, lysophospholipids, and DGs.

**FIGURE 8:**
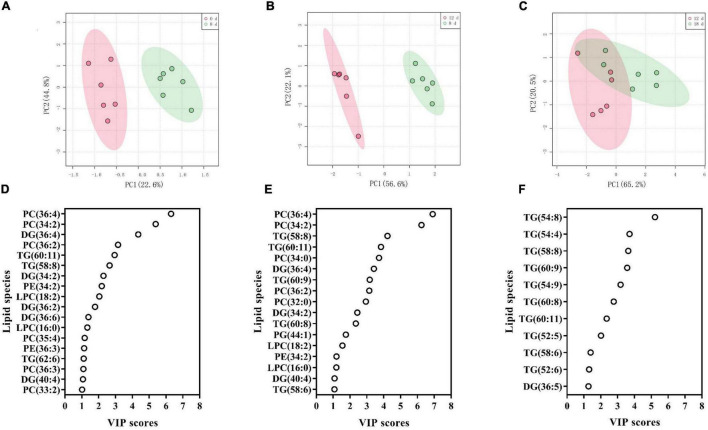
**(A–C)** PLS-DA score plots demonstrating the differences between adjacent aging time points: **(A)** 0 d vs. 8 d, **(B)** 8 d vs. 12 d, and **(C)** 12 d vs. 18 d. **(D–F)** PLS-DA VIP scores identifying critical lipid species (VIP > 1 and *p* < 0.05) that differentiated between treatments: **(D)** 0 d vs. 8 d, **(E)** 8 d vs. 12 d, and **(F)** 12 d vs. 18 d. Lipid molecular species are identified as total acyl carbons: total double bonds.

To identify the crucial lipids contributing to the differences between the adjacent aging time points, we utilized PLS-DA to visualize the sample distribution according to the 223 lipid species (Figure 8 and Supplementary Table 4). The samples from 0 d, 8 d, and 12 d after the aging treatment were largely separated from each other, according to the PLS-DA score plots (Figures 8A,B), while the scatter plot of the 12-d group was more similar to that of the 18-d group (Figure 8C), which is consistent with the results in Figure 7. The critical lipid species were ranked based on their ability to distinguish the samples; 18, 17, and 11 lipid species were identified for the 0 d vs. 8 d, 8 d vs. 12 d, and 12 d vs. 18 d comparisons, respectively (Figures 8D–F). During the early stage of seed aging, 10 of the 18 lipids were PC, LPC, or PE species (Figure 8D). However, no phospholipid species were found during the later aging period (Figure 8F). These results indicated that the changes in lipids differed between the 8 d and late stage of seed aging. Therefore, the 11 shared lipid species from 0 d to 8 d and 8 d to 12 d were chosen as possible critical lipids, including DG 34:2, 36:4, and 40:4, LPC 16:0, and 18:2, PC 34:2, 36:2, and 36:4, PE 34:2, and TG 58:8 and 60:11.

**FIGURE 9:**
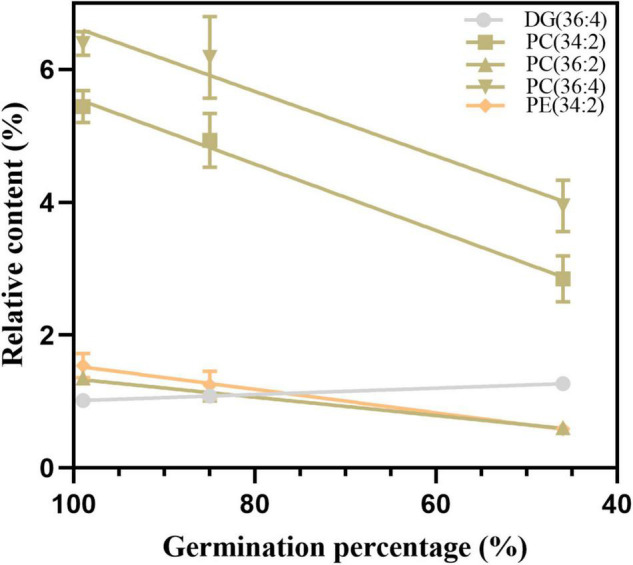
The changes in five candidate lipids after 24 h of imbibition. Data shows the mean ± SD of six biologically independent experiments. Lipid molecular species are identified as total acyl carbons: total double bonds.

To confirm the reliability of the index, the 11 possible critical lipids were detected in the seeds after 24 h of imbibition, and only 5 lipids were detected including (DG 36:4, PC 34:2, 36:2, and 36:4, and PE 34:2) (Figure 9). In contrast to the change in DG 36:4, all phospholipids steadily reduced along with seed aging. Moreover, the correlation analysis showed that seed viability and lipids were significantly correlated (Supplementary Table 5). These findings were in agreement with the changes in lipids in aged seeds without imbibition.

## Discussion

The excessive accumulation of ROS and lipid oxidation during seed aging have been postulated to be the primary causes of seed deterioration and death (Tian et al., 2008; Ouzouline et al., 2009; Hu et al., 2012; Ratajczak et al., 2015). Here, we investigated the activity of antioxidant systems, ROS accumulation, cellular membrane damage, and phospholipid metabolism associated with the loss of seed viability in soybean.

The key to ensuring seed viability is the effective maintenance of redox homeostasis. The antioxidant system plays a vital role in maintaining ROS balance and cellular homeostasis. CAT and SOD are the primary enzymes that prevent the excessive accumulation of O_2_^–^ and H_2_O_2_ (Bailly and Bailly, 2004). Additionally, in the AsA-GSH cycle, APX, DHAR, MDHAR, and GR, which are associated with low-molecular-weight non-enzymatic antioxidants, such as AsA and GSH, also participate in H_2_O_2_ scavenging (Kurek et al., 2019). APX, CAT, GR, and SOD activities declined in cotton seeds during seed aging (Goel and Sheoran, 2003). When aging wheat seeds, the activities of CAT and SOD decreased, while an increase in GR activity was detected (Lehner et al., 2008). In aged rice seeds, there was a significant decrease in the activity levels of APX, CAT, and MDHAR, while the activities of DHAR, GR, and SOD remained constant (Yin et al., 2014). In the present study, we observed a higher SOD activity level after seed aging, while CAT activity declined significantly. In contrast, enzyme activity from the AsA-GSH cycle remained nearly stable. These results demonstrated a relationship between enzymatic activity and seed viability, and the mechanism of antioxidant enzymes in response to aging stress differs among species. According to our previous study, the activity levels of SOD and the AsA-GSH cycle enzymes isolated from mitochondria were significantly reduced in aged soybean seeds (Xin et al., 2014). This observation implies that mitochondria are particularly vulnerable to oxidative stress. In addition, the decrease in CAT activity from multiple species indicates that CAT may be an important antioxidant enzyme in seed aging (Kibinza et al., 2011). Compared with the AsA-GSH cycle enzymes, antioxidant content, such as that of AsA and GSH, declined sharply in the 8 d-aged seeds, which indicated that the cellular redox environment transitions to the oxidized state. Therefore, low-molecular-weight antioxidants are critical factors during the early stage of seed viability loss in soybean. Furthermore, both the GSSG and reduced GSH contents decreased dramatically after the aging treatment. The size of the GSH pool and its reduction play a critical role in cell division, growth, and apoptosis (Noctor et al., 2012). One explanation for this phenomenon may be that GSH reacts with protein cysteine residues to form *S*-glutathionylated proteins, which can prevent irreversible oxidation (Dalle-Donne et al., 2007).

Mitochondria, peroxisomes, endoplasmic reticula, and plasma membrane NADPH oxidases are the primary sources of ROS (Sharma et al., 2012; Kijowska-Oberc et al., 2021). Despite their toxic effects, ROS are well-known second messengers in a variety of biological processes, including the alleviation of dormancy, enhanced rapid seed germination, and environmental stress tolerance (Bailly et al., 2008; You and Chan, 2015). SOD catalyzes the dismutation of O_2_^–^ to H_2_O_2_. H_2_O_2_ is not highly reactive; however, its half-life is long, and it can further form the hydroxyl radical (OH⋅), which is the most aggressive form of the oxygenated derivatives. In the present study, the peak O_2_^–^ and H_2_O_2_ contents were observed at 12 d of aging, which is consistent with previous studies (Tian et al., 2008; Xin et al., 2014; Xia et al., 2015). However, the decrease in ROS in the late stage of seed aging may have been affected by the reduction in cellular metabolic activity, particularly by the degradation of mitochondria (Xin et al., 2014; Xia et al., 2015; Yin et al., 2016; Mao et al., 2018). Compared with the synergistic changes of the superoxide anion and SOD, strictly limiting the H_2_O_2_ content should be the focus of further research. Electrolyte leakage is primarily affected by changes in the membrane structure and phospholipid composition, and it is used as a crucial indicator of cell membrane function and permeability (Ratajczak et al., 2015). A decrease in the integrity of the plasma membrane reduces cell viability (Schapire et al., 2008). Significantly higher electrolyte leakage rates were observed in the seeds aged 8 d and 18 d than in the other seeds; this result is related to changes in the structure of the membrane and the composition of lipids during seed preservation. ROS accumulation and membrane damage can alter the proton concentrations and decrease the pH (Nagel et al., 2019); however, how these changes in the intracellular environment affect seed viability remains unclear. MDA is the end-product of membrane lipid peroxidation and reflects the degree of lipid peroxidation (Li et al., 2018). In this study, a significant increase in MDA content was observed after aging seeds for 12 d. This result indicates that membrane lipid peroxidation occurs during seed aging, but it is not the primary factor for the early stage of seed viability loss. The synergistic change in the MDA and ROS content indicated that lipid peroxidation was instrumental in in the later stage of seed aging. Therefore, changes in membrane structure and function, represented by an increase in electrolyte leakage, are more closely related to seed viability.

To understand the changes in the membrane structure and explore the dynamics of the membrane lipid metabolism during seed aging, we used the lipidomic method. Although seed aging was accompanied by phospholipid degradation, the change patterns of phospholipids in this study were different from those reported in previous studies (Powell and Matthews, 1981; Pukacka and Kuiper, 1988; Tanida, 1996; Ouzouline et al., 2009; Lee et al., 2012; Wang et al., 2012; Riewe et al., 2017; Chen et al., 2022). In this study, the PA, PC, PE, PI, and PS contents substantially decreased, while the PG content increased, indicating that the changes in phospholipid metabolism during seed aging vary among crop species and treatment conditions. However, the phospholipid metabolism results were not consistent with the changes in the electrical conductivity. Membrane lipids can be converted into TG and DG under heat stress (Légeret et al., 2016). The accumulation of TGs from membrane lipids degradation can maintain *Arabidopsis* survival in extended darkness (Fan et al., 2017) and enhance the tolerance of the plant to chilling, freezing, and heat stress (Shimada et al., 2008; Arisz et al., 2018; Higashi and Saito, 2019). Similarly, the DG and TG contents increased significantly after aging treatment, which indicated that DG and TG may serve as buffer zones against lipotoxicity. Riewe et al. (2017) also reported that the hydrolytic cleavage of oxidized phospholipids resulted in the formation of oxidized lysophospholipids and oxidized DGs, and the reduction of phospholipids was inconsistent with the accumulation of their oxidation products. In the current study, a similar decline range was found among PA, PC, and PE, while the contents of LPA and LPC increased more than that of LPE. One explanation for this is that phospholipase C (hydrolyzing phospholipids to form the DG) may preferentially degrade PE (Peters et al., 2014; Pejchar et al., 2015), and the phospholipids may be oxidized during seed aging (Bayon et al., 2015). Our data also do not support a significant role for phospholipase D which converts phospholipids to PAs during seed aging (Devaiah et al., 2007; Lee et al., 2012; Wiebach et al., 2020).

Changes in membrane structure greatly affect the function and characteristics of cell membranes (Upchurch, 2008). Membrane lipid unsaturation was found to increase to resist salt- and chilling-induced injuries (Elkahoui et al., 2004; Bakht et al., 2006; Zheng et al., 2016), while it decreased to tolerate heat stress (Falcone et al., 2004). An increase in the ACL of phospholipids can lead to lower membrane fluidity (Bach and Faure, 2010). Compared with the increase in the PC and total phospholipid DBIs in the present study, the DBIs of PE, PG, PI, and PS decreased after seed aging. Wada and Murata (2007) indicated that high amounts of saturated PGs can decrease membrane fluidity. Surprisingly, no changes in the DBIs of the phospholipids were detected in the early stage of seed aging. The ACL of different phospholipids was observed to increase significantly, and an increase in total phospholipids was found at 12 d of aging treatment. In a previous study, the ACL of the PS was considered as an indicator for assessing plant senescence (Li et al., 2014). According to these data, we can infer that the DBI and ACL of phospholipids affect the fluidity and stability of the membrane system after 8 d of aging treatment. The rapid increase in the PC/PE ratio at 8 d indicated that unstable phospholipids were preferentially hydrolyzed, which may be beneficial for maintaining the stability of the membrane system in the early stage of seed aging.

Furthermore, we used the PCA method to explore the contribution of lipid changes to sample differences. The degradation of phospholipids and accumulation of lysophospholipids and DGs indicated that membrane lipid changes are related to the early stage of seed viability loss. We further explored the critical lipids between different time points, and the results demonstrated that phospholipid degradation and glycerolipid synthesis are the primary events in seed aging. Several phospholipids decreased at approximately 80% germination, such as PC 36:3 and PE 36:3, which have been observed in previous studies (Lee et al., 2012; Wang et al., 2012; Oenel et al., 2017). In this study, we identified five lipids (DG 36:4, PC 34:2, 36:2, and 36:4, PE 34:2) as new indicators for seed viability detection, and their reliability was evaluated in imbibed seeds after their aging.

**FIGURE 10:**
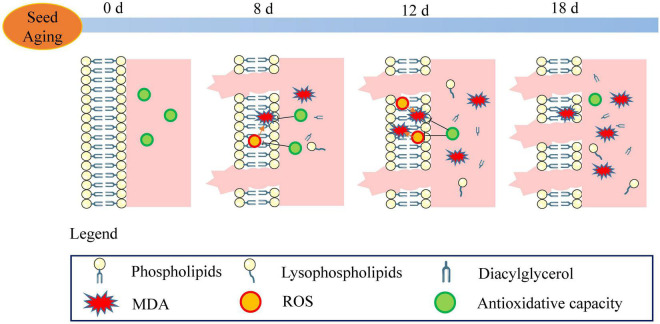
Schematic of the metabolic events occurring during seed aging. The stable membrane structure and high antioxidant capacity were observed in the unaged seeds (aged for 0 d). With the increase in ROS content and the decrease in antioxidant capacity, lipid peroxidation (MDA) and electrolyte leakage were enhanced. The phospholipid degradation and the increase in lysophospholipid and diacylglycerol were observed in the 8 d-aged seeds. ROS steadily increased along with seed aging (12 d) leading to a decrease in the antioxidant capacity and an increase in the MDA content. Compared to the reduction in the phospholipid content, the content of lysophospholipid and diacylglycerol increased gradually. In the 18 d-aged seeds, ROS content decreased and electrolyte leakage increased. In the course of seed aging, these events result in the loss of seed viability.

A schematic of the metabolic events occurring in the aged soybean seeds is displayed in Figure 10. The excessive accumulation of ROS and decreased antioxidant capacity in artificially aged soybean seeds disrupted cellular redox homeostasis, resulting in lipid peroxidation, reduced phospholipid content, aggravated cell membrane injury, and destroyed membrane permeability and stability. Further, the function of the membrane system was disordered, and seed viability was reduced. Moreover, the results implied that the enhanced turnover of phospholipids to glycerolipids and lysophospholipids may serve as a critical pathway in the response to seed aging. Although the present study does not support that the degradation of phospholipids through phospholipase D is the cause of seed viability decline (Devaiah et al., 2007; Lee et al., 2012), maintaining membrane integrity and stability may play a crucial role in enhancing seed viability.

## Conclusion

The study results demonstrated that seed viability is strongly associated with the membrane lipid metabolism in soybean after seed aging. ROS accumulation and antioxidative capacity decline led to increased electrolyte leakage and MDA, which confirmed that non-enzymatic antioxidants are more critical for maintaining intracellular homeostasis for seed viability. Furthermore, a significant decrease in PA, PC, PE, PS, PG, PI, and LPG and a significant increase in DG, PG, LPA, LPC, LPE, and TG during seed aging indicated that phospholipid hydrolysis, which formed glycerolipids and lysophospholipids, may be caused by oxidative stress. Moreover, the ratio of PC/PE increased, and the DBI and ACL of the phospholipids remained stable after slight aging, demonstrating that maintaining the structural stability of phospholipids favors phospholipid membrane function and seed viability. Furthermore, we chose DG 36:4, PC 34:2, 36:2, and 36:4, and PE 34:2 as novel molecular indicators for seed viability. In conclusion, membrane lipid metabolism plays a crucial role in response to aging in soybean seeds. In the future, we will conduct an in-depth exploration of the molecular regulatory mechanisms of lipid peroxidation and phospholipids during seed aging.

## Data Availability Statement

The original contributions presented in this study are included in the article/Supplementary Material, further inquiries can be directed to the corresponding author/s.

## Author Contributions

XX, G-KY, Y-CZ, and X-XL designed the research. Y-XL and H-JX performed experiments. Y-XL, H-JX, and XX analyzed the results. Y-XL and XX wrote the manuscript. X-XL, Y-CZ, and G-KY helped with editing the manuscript before submission. All authors read and agreed with the final version of the manuscript.

## Conflict of Interest

The authors declare that the research was conducted in the absence of any commercial or financial relationships that could be construed as a potential conflict of interest.

## Publisher’s Note

All claims expressed in this article are solely those of the authors and do not necessarily represent those of their affiliated organizations, or those of the publisher, the editors and the reviewers. Any product that may be evaluated in this article, or claim that may be made by its manufacturer, is not guaranteed or endorsed by the publisher.

## Footnotes

1
http://soystats.com

2
http://www.lipidmaps.org

3
https://www.metaboanalyst.ca/
